# Ophthalmological Manifestations of Hereditary Amyloidosis due to Transthyretin: A Systematic Review

**DOI:** 10.1155/joph/5277348

**Published:** 2026-04-30

**Authors:** Maria Luisa Fialho, Claudia Pedreira, Juliana Marback, Tonnison Silva, Marcela Costa, Luiz Eduardo Ritt

**Affiliations:** ^1^ Student Department, Bahiana School of Medicine and Public Health, Salvador, Bahia, Brazil, bahiana.edu.br; ^2^ Ophthalmology Department, Santo Antônio Hospital, Associação Obras Sociais Irmã Dulce, Salvador, Bahia, Brazil; ^3^ Cardiology Department, Hospital Cardio Pulmonar, D’Or Institute of Research and Teaching, Salvador, Bahia, Brazil; ^4^ Neurology Department, Hospital Cardio Pulmonar, D’Or Institute of Research and Teaching, Salvador, Bahia, Brazil

**Keywords:** amyloidosis, familial amyloid neuropathy, ophthalmology, proalbumin, transthyretin

## Abstract

**Introduction:**

Amyloidosis involves the deposition of fibrillar proteins in tissues. Hereditary transthyretin amyloidosis (ATTRv), caused by TTR gene mutations, affects various tissues, with ocular involvement in about 10% of patients.

**Objective:**

To describe and quantify ocular manifestations in ATTRv through a systematic review.

**Methods:**

We reviewed studies on ocular manifestations of ATTRv, including only articles in Portuguese and English, excluding case reports, conference abstracts, and duplicates.

**Results:**

Sixteen studies with 1792 patients were analyzed. Vitreous opacities were found in 87.5% of studies (36% frequency). Dry eye was reported in 50% (48.5% frequency) and scalloped pupil in 37.5% (35.9% frequency). Glaucoma had an average frequency of 18.2%, while amyloid deposits in the lens appeared in 37.5% of studies (25.8% frequency). Retinal hemorrhage and vascular tortuosity were less common, and conjunctival lymphangiectasia was found in one study (54.2%). The Val30Met mutation was most prevalent, noted in 92% of cases. No clear link between ocular and systemic symptoms was identified.

**Conclusion:**

Vitreous opacities were the most common ocular manifestation, followed by dry eye and scalloped pupil. Further research is needed due to the limited number of representative studies.

## 1. Introduction

Amyloidosis is a disease characterized by the deposition of insoluble proteins and misfolded fibrillar aggregates—amyloid deposits—in tissues [1–4]. This is primarily due to a genetic mutation but can also result from inflammatory, degenerative, and neoplastic processes [5]. Depending on the type of amyloidosis, various factors can be responsible for protein aggregation [6].

Hereditary amyloidosis has three main forms, according to the amyloid precursor protein: transthyretin (TTR), which accounts for over 95% of amyloidosis cases [7, 8]; apolipoprotein A1 and gelsolin [1]. The synthesis of TTR primarily occurs in the liver, which accounts for about 90% of its production, as well as in retinal pigment epithelial (RPE) cells and the choroid plexus of the brain [5].

Hereditary ATTR (ATTRh or ATTRv) is a rare, progressive, multisystemic, and disabling autosomal dominant polyneuropathy characterized by the accumulation of TTR amyloid deposits in the autonomic and peripheral nervous systems [9, 10]. The diagnosis is based on genetic testing for the presence of pathogenic variants in the TTR gene and the identification of amyloid deposits in a tissue sample, most commonly from the salivary gland, abdominal fat, rectum, heart, or peripheral nerve [5].

The first identified mutation was Val30Met (later described as Val50Met due to a change in nomenclature) [11], which remains the most common mutation. The main hotspots of the disease are in Europe—particularly in Portugal and Sweden—and Japan, but the distribution of ATTR is widespread. Penetrance and expressivity vary depending on the country, mutations, and ethnic groups [12].

Although clinical manifestations are more predominant in the peripheral nerves and heart, the eye is a potential target organ, affecting about 10% of patients with ATTRv amyloidosis in the later stages of the disease [5, 9]. Proteins that can form amyloid deposits involving the eye include TTR and gelsolin, where ocular involvement is part of a systemic disease, as well as keratoepithelin and lactoferrin, where the pathological process is localized exclusively in the eye.

Ophthalmological findings vary depending on the specific mutation presented, which can include abnormal tear film breakup time and Schirmer test, amyloid deposition in the iris and anterior capsule of the lens, shell‐shaped iris, glaucoma, vitreous amyloidosis, abnormal conjunctival vessels, and retinal amyloid angiopathy [13].

Studies indicate that amyloid deposition in the lacrimal glands occurs at advanced stages; however, the early presence of the condition suggests mechanisms beyond amyloid deposits. Pupil anomalies, such as “shell‐shaped pupil,” “bilateral Horner’s syndrome,” and “tonic pupils,” are common manifestations attributed to parasympathetic or muscular involvement. Vitreous opacity, typically bilateral and asymmetric, is the most common ophthalmological alteration in ATTRv Val30Met amyloidosis, presenting various types of deposits, including lenticular pseudopodia, fibrils, and spherical opacities. Chronic open‐angle glaucoma is a serious complication, varying in prevalence and associated with rapid progression to visual loss. Vascular abnormalities in the eye, affecting the conjunctiva, retina, and choroid, are frequent, with retinal diseases being less common than conjunctival disorders. Amyloid deposits around the retinal and choroidal vessels are widespread and may be present even in asymptomatic individuals. In mutations with predominant cardiac involvement, retinal microangiopathy with retinal vasculitis leading to retinal ischemia and vitreous hemorrhage has been described. Vascular disease in ATTR can cause macular edema and optic disc swelling, even in patients without vitreous involvement, as well as secondary neovascular glaucoma [13].

Existing therapies, such as liver transplantation, TTR stabilizers, and RNA silencers, which modify the natural course of the disease, are unable to prevent intraocular amyloid deposition [14]. Thus, the ophthalmological manifestations of ATTRv benefit from treatments tailored to each specific alteration expressed [5]. Despite some studies reporting that tafamidis, although it does not prevent the progression of ocular disease, may be involved in reducing its severity [13].

The ophthalmologist plays a crucial role in the suspicion, diagnosis, and management of these diseases [5]. Moreover, it is important to note that patients with ATTRv, with or without liver transplantation, should undergo regular ophthalmological follow‐up due to the extra‐hepatic sites of TTR production. Periodic consultations have been suggested starting from the time of diagnosis. The initial consultation would be in this context, with subsequent visits depending on the patient’s clinical condition: repeated evaluations every 2 years for asymptomatic patients or annually for symptomatic ones [13].

Given the diagnostic challenges and the progressive, debilitating nature of this disease, this study aims to describe the frequency and distribution of ophthalmological alterations presented by patients with this subtype of amyloidosis, with the goal of facilitating the identification of these alterations and thus optimizing treatment. Additionally, the secondary objective is to describe the correlation between ophthalmological and systemic manifestations (namely, polyneuropathy and cardiomyopathy) in patients with ATTRv.

## 2. Methods

This is a systematic literature review.

### 2.1. Study Question

The study question was designed according to the PECO strategy (an acronym for: population, exposure, control, and outcome). The defined population consisted of patients with amyloidosis; the exposure was the presence of a mutation in the TTR gene; the control group included patients without amyloidosis; and the outcome was the presence of ophthalmological manifestations.

The inclusion criteria were clinical studies on ophthalmological manifestations in patients with any variant of ATTRv, published in any time frame and in English or Portuguese.

Regarding the exclusion criteria, studies addressing ophthalmological manifestations of TTR amyloidosis in its nonhereditary form or light chain amyloidosis (AL) form were excluded, as well as those lacking full texts in the specified languages and duplicates found in the search. Additionally, systematic reviews, literature reviews, and case reports with fewer than 10 patients, as well as abstracts submitted to congresses, were excluded.

### 2.2. Search Strategy

The search strategy employed was as follows: (“amyloidosis familial” OR “Amyloidosis” OR “amyloidosis”) AND (“Hereditary” OR “amyloidosis hereditary” OR “amyloidosis, familial” OR “transthyretin amyloidosis” OR “Hereditary” OR “prealbumin” OR “Transthyretin” OR “transthyretins”) AND (“Ocular manifestations” OR “Glaucoma” OR “Cataracts” OR “Lens opacity” OR “Vitreous opacity” OR “Pupil abnormality” OR “Dry eyes” OR “keratoconjunctivitis sicca”).

The variables used were TTR; familial amyloid polyneuropathy (independent variables); and ophthalmological involvement, vitreoretinal changes, glaucoma, cataract, dry eye, and keratopathy (dependent variables).

The complete search strategy is listed in Table 1 of Supporting Information—Detailed Search Strategy.

### 2.3. Study Selection

A search was conducted in the databases PubMed, Embase, Web of Science, Scopus, SciELO, and Cochrane using the detailed search strategy. Additionally, all researchers performed a manual search for relevant articles based on the references of other studies.

The titles identified through the search strategy and manual search were initially screened by analyzing their titles. Subsequently, the abstracts of the selected studies were reviewed, with another round of screening based on the inclusion and exclusion criteria defined. Finally, the full texts of the articles were read, resulting in the final group of selected studies. All steps were carried out by two reviewers independently. A third reviewer was involved when there was disagreement regarding the inclusion or exclusion of titles.

### 2.4. Data Extraction

For each study selected for inclusion in the systematic review, the following data were extracted: the name(s) of the author(s) and the year of publication; the journal in which it was published; the sample size; and other relevant information, such as the total number of patients evaluated in each study, the sex distribution of these individuals, and their countries of origin.

The extracted data were summarized in a clear and concise descriptive format in tables presented in the results section and throughout the text. To facilitate understanding and visualization, the results were presented in the form of tables and graphs, generated using the open‐source software RStudio with R programming language version 4.4.0.

### 2.5. Risk of Bias Analysis

In the risk of bias analysis, an adaptation of the Newcastle–Ottawa Scale (NOS) was used, along with the criteria of Pierson and Bradford Hill [15], to assess the methodological quality of the studies included in this systematic review. The criteria were grouped into the same four domains utilized in the NOS: selection, determination, causality, and reporting. This approach was deemed more appropriate due to the heterogeneity of the included studies, which often did not fit the predefined case‐control or cohort classifications established by the NOS.

Instead of assigning an aggregate score, a global assessment of methodological quality was conducted, considering the most critical criteria for the validity of each study within the specific clinical context. Additionally, as suggested by the authors [15], issues more focused on studies evaluating adverse effects were not taken into consideration, as this type of study was not included in this review.

Studies with a low risk of bias were characterized by the exclusive presence of negative or not applicable responses for the evaluated parameters. In contrast, studies with a medium risk presented at least one negative response or a combination of a negative response and another not applicable, while high‐risk studies showed multiple negative responses.

The analysis was conducted by two reviewers independently, and any discrepancies were resolved by consensus or by consulting a third reviewer if necessary.

The results of the bias risk analysis were presented in a summarized and critical manner in the form of a table, highlighting the main sources of bias in the included studies and discussing their potential impact on the results of the systematic review.

## 3. Adherence to Ethical Standards in Systematic Reviews

This systematic review has been conducted in strict accordance with ethical standards, ensuring that all methodological procedures are in line with established ethical guidelines. The systematic review process involves a comprehensive and objective synthesis of existing literature and does not involve direct interaction with human subjects or the collection of primary data. As such, the nature of this study inherently avoids ethical concerns typically associated with research involving human participants.

Due to its intrinsic characteristics, including the reliance solely on publicly available data and previously published research, this systematic review did not necessitate submission to an ethics committee. The review process adhered to ethical principles by ensuring the integrity of the data, transparency in methodology, and proper citation of sources. This approach maintains the highest standards of research ethics while effectively addressing the research question through rigorous and ethical review of the existing literature.

## 4. Results

### 4.1. Study Selection

**FIGURE 1:**
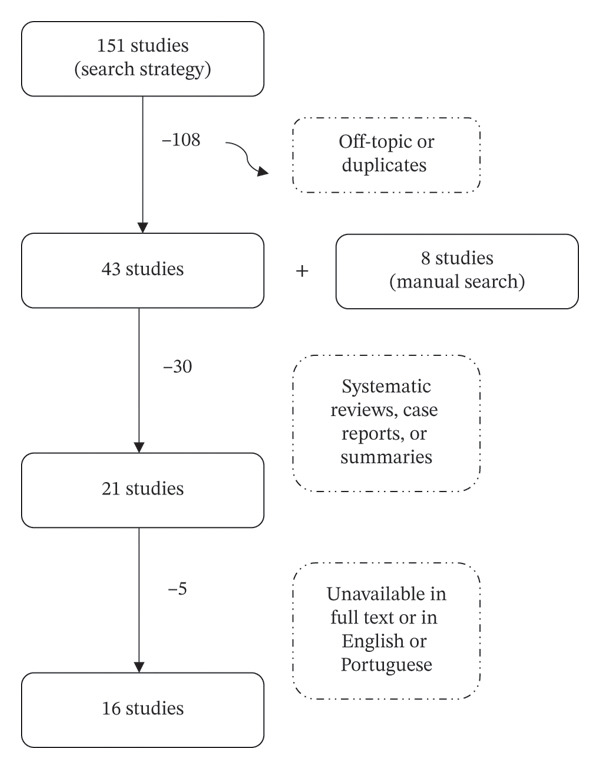
Diagram flow of data selection.

The SciELO and Cochrane databases did not produce relevant results regarding the specific research question. Therefore, the studies selected for analysis in this work were those obtained from the PubMed, Embase, Web of Science, and Scopus databases, as well as those found through manual search. Figure 1 below illustrates the application of the inclusion and exclusion criteria used to obtain the studies addressed in this work.

A total of 151 studies were collected from the described search strategy. Of these, 108 were excluded based on their titles for not fitting the theme of this work or being identified as duplicates among results from different databases. The remaining 43 studies were supplemented by 8 found through manual search based on title analysis. From the total of 51 studies at this stage, 30 were excluded, including systematic reviews, case reports, and abstracts from conference presentations, after reviewing their abstracts. Finally, after reading the full texts, 5 studies were excluded from the remaining 21 due to unavailability in full text in Portuguese or English, resulting in a total of 16 studies included in this work.

### 4.2. Data Extraction

Table 1 presents the list of the sixteen articles used to extract the results for this work, including the title, authors’ names, year of publication, and journal of publication.

**TABLE 1:** General data.

Paper	Title	Journal	Year
Ando E, Ando Y, Okamura R, Uchino M, Ando M, Negi A.	Ocular manifestations of familial amyloidotic polyneuropathy type I: long term follow up	Br J Ophtalmol	1997
Beirão JM, Malheiro J, Lemos C, et al.	Impact of liver transplantation on the natural history of oculopathy in Portuguese patients with transthyretin (V30M) amyloidosis	Amyloid	2014
Beirão JM, Malheiro J, Lemos C, Beirão I, Costa P, Torres P.	Ophthalmological manifestations in hereditary transthyretin (ATTR V30M) carriers: a review of 513 cases	Amyloid	2015
Bunod R, Adams D, Cauquil C, et al.	Conjunctival lymphangiectasia as a biomarker of severe systemic disease in Ser77Tyr hereditary transthyretin amyloidosis	Br J Ophtalmol	2019
Buxbaum JN, Brannagan T, Buades‐Reinés J, et al.	Transthyretin deposition in the eye in the era of effective therapy for hereditary ATTRV30M amyloidosis	Amyloid	2019
Choi KJ, Son KY, Kang SW, Kim D, Choi JO, Kim HJ.	Asp38Ala and Thr59Lys Familial transthyretin amyloidosis	RETINA	2022
Kawaji T, Ando Y, Ando E, Sandgren O, Suhr OB, Tanihara H.	Transthyretin‐related vitreous amyloidosis in different endemic areas	Amyloid	2010
Kimura A, Ando E, Fukushima M, et al.	Secondary glaucoma in patients with familial amyloidotic polyneuropathy	Arch Ophtalmol	2003
Liu T, Zhang B, Jin X, et al.	Ophthalmic manifestations in a Chinese family with familial amyloid polyneuropathy due to a TTR Gly83Arg mutation	Eye	2014
Long D, Zeng J, Wu LQ, Tang LS, Wang HL, Wang H.	Vitreous amyloidosis in two large mainland Chinese kindreds resulting from transthyretin variant Lys35Thr and Leu55Arg	Ophthalmic Genet	2012
Marques JH, Coelho J, Malheiro J, Pessoa B, Beirão JM.	Subclinical retinal angiopathy associated with hereditary transthyretin amyloidosis—assessed with optical coherence tomography angiography	Amyloid	2020
Ohya Y, Okamoto S, Tasaki M, et al.	Manifestations of transthyretin‐related familial amyloidotic polyneuropathy: long term follow‐up of Japanese patients after liver transplantation	Surg Today	2011
Reynolds MM, Veverka KK, Gertz MA, et al.	Ocular manifestations of familial transthyretin amyloidosis	Am J Ophtalmol	2017
Rousseau A, Terrada C, Touhami S, et al.	Angiographic signatures of the predominant form of familial transthyretin amyloidosis (Val30Met mutation)	Am J Ophtalmol	2018
Ruiz‐Medrano J, Puertas M, Almazán‐Alonso E, et al.	Ophthalmology involvement in patients with hereditary transthyretin amyloidosis	Retina	2023
Zou X, Dong F, Zhang S, Tian R, Sui R.	Transthyretin ALA36Pro mutation in Chinese pedigree of familial transthyretin amyloidosis with elevated vitreous and serum vascular endothelial growth factor	Exp Eye Res	2013

A total of 1792 patients were evaluated, with the studies by Buxbaum et al. [16] and Beirão et al. [17] having the largest number of participants (804 and 513, respectively). Additionally, the former does not report the number of patients by the described location. The average number of patients, excluding these two cited articles, is approximately 34 patients per study.

### 4.3. Risk of Bias Analysis

Of the 16 studies evaluated, 2 (12.5%) were identified as high risk, indicated in Table 2 with red markers, while 7 (43.75%) were classified as medium risk, highlighted in yellow. The remaining 7 studies were considered low risk, marked in green.

**TABLE 2:** Risk of bias analysis.

	Ando et al. [**18**]	Kimura et al. [**19**]	Kawaji et al., [**20**]	Ohya et al., [**21**]	Long et al., [**22**]	Liu et al., [**23**]	Zou et al., [**24**]	Beirão et al., [**25**]	Beirão et al., [**17**]	Reynolds et al., [**26**]	Rousseau et al., [**27**]	Bunod et al., [**28**]	Buxbaum et al., [**16**]	Marques et al., [**29**]	Choi et al., [**30**]	Ruiz‐Medrano et al., [**31**]

*Selection*																
Representativeness of cases	YES	YES	YES	NO	NO	NO	NO	YES	YES	YES	YES	YES	YES	YES	YES	YES

*Ascertainment*																
Was the exposure adequately ascertained?	YES	YES	YES	YES	YES	YES	YES	YES	YES	YES	YES	YES	YES	YES	YES	YES
Was the outcome adequately ascertained?	YES	YES	YES	YES	NO	YES	NO	YES	YES	YES	YES	YES	YES	YES	YES	YES

*Causality*																
Was the follow‐up long enough for outcomes to occur?	NO	NO	NO	YES	NO	NO/NA	NO/NA	NO	YES	YES	NO/NA	NO/NA	NO	NO/NA	NO/NA	NO/NA

*Reporting*																
Is the article described with sufficient details to allow others to replicate the research?	YES	YES	YES	YES	YES	YES	NO	YES	YES	YES	YES	YES	YES	YES	YES	YES

*Overall*	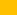	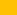	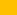	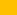	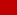	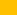	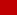	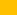	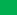	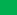	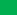	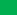	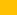	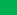	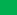	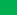

*Note:* N/A: not applicable; classified as such for cases where the studies did not have patient follow‐up due to their design.

While low‐risk studies provide greater confidence in the integrity of the presented results, medium and high‐risk studies require more critical analysis, considering the potential impact of bias on the validity of the findings.

### 4.4. Summary of Individual Study Results

#### 4.4.1. Distribution of TTR Gene Mutations

The most common mutation observed across the results of the articles was Val30Met, representing approximately 92% of the observations (1643 cases) from the analyzed studies.

Regarding the distribution of variants, Table 3 presents the breakdown of the type of mutation and the number of affected patients for each study.

**TABLE 3:** Types of TTR mutations present by study.

Paper	TTR variant (*n*)
Ando et al. [18]	Val30Met (37)
Beirão et al. [25]	Val30Met (64)
Beirão et al. [17]	Val30Met (513)
Bunod et al. [28]	Ser77thr (24)
Buxbaum et al. [16]	Val30Met (804)
Choi et al. [30]	Asp38Ala (9), Glu89Lys (3), Thr59Lys (2) Met13Ile (1), Lys35Asn (1)
Kawaji et al. [20]	Val30Met (90)
Kimura et al. [19]	Val30Met (41), Tyr114Cys (6), Ser50Ile (1), Val30Met + Arg104His (1)
Liu et al. [23]	Gly83Arg (8)
Long et al. [22]	Lys35Thr (8), Leu55Arg (8)
Marques et al. [29]	Val30Met (24)
Ohya et al. [21]	Val30Met (30), Tyr114Cys (3), Ser50Ile (1)
Reynolds et al. [26]	Ala12Ser (1), Ala45Asp (2), Asp38Ala (1), Glu74Ser (1), Gly47Glu (2), Ile107Val (1), Ile84Thr (1), Leu58His (3), Phe33Leu (1), Ser77Thr (3), Thr60Ala (9), Thr69His (1), Val122Ile (6), Val30Met (14), Ala36Pro (1), Asp18Glu (1), Glu54Gly (1), Glu89Lys (1), Gly47Arg (1), Gly6Ser (1), Phe33Ile (1), Tyr114Cys (1)
Rousseau et al. [27]	Val30Met (18)
Ruiz‐Medrano et al. [31]	Val30Met (8), Val122Ile (8), Glu89Lys (7), Ser23Asn
Zou et al. [24]	Ala36Pro (11)

#### 4.4.2. Ocular Clinical Manifestations

The ophthalmological findings identified in the studies were compiled and are reported in terms of frequency, indicating the percentage of patients who exhibited specific ophthalmological manifestations relative to the total number of study participants. Some studies did not provide results for certain observations, as they were not the focus of the study in question and, consequently, are not included in the summary of results. When the authors reported that no patients in the study exhibited the manifestation of interest, the article is presented in the results with a frequency of zero.

#### 4.4.3. Vitreous Opacities

There are studies where no patients exhibited this alteration, such as Marques et al. [29], while in others, it was found in 100% of patients, as in the case of Liu et al. [23]. In total, fourteen articles (87.5%) assessed the presence of vitreous opacities.

The average frequency of this finding (37.3%) does not accurately reflect the distribution of the data due to the significant variability among the studies, as indicated by the standard deviation of 32.1%.

Kawaji et al. [20] report that vitreous opacities were a criterion for patient inclusion in the study, whereas Buxbaum et al. [16] presented results indicating severe ocular anomalies, which included vitreous opacities, but did not provide individual values necessary for calculating frequency.

#### 4.4.4. Dry Eye

The presence of dry eye was reported in 8 studies (50%), with an average frequency of 47.8%. However, this average was significantly influenced by the studies conducted by Reynolds et al. [26] and Rousseau et al. [27], which presented results that were inconsistent with those of the other studies, as reflected by a standard deviation of 33.4%.

Among the evaluated articles, the manifestation of dry eye was assessed using Schirmer’s test and the break‐up time (BUT), or even both tests in conjunction, as seen in Beirão et al. [25].

#### 4.4.5. Pupil Alterations

The pupillary manifestation assessed in the studies was the “scalloped pupil,” reported by six authors (37.5%). The results indicate a frequency below 50% in most studies, with the only exception being the study by Marques et al. [29], where the presence of this manifestation was a requirement for participation due to the authors’ research objectives. The average frequency of a scalloped pupil was 35.5%, with a standard deviation of 33.6%.

#### 4.4.6. Glaucoma

This manifestation was reported by ten out of sixteen authors (62.5%). The average frequency for this manifestation was 18%, with a standard deviation of 15.7%, with the highest frequency occurring in the study by Ohya et al. [21], which reported 52.9% of affected patients.

It is also worth noting that Ohya et al., [21] observed glaucoma progression in 9 out of 17 patients, although they did not provide information regarding the prior existence of this manifestation in the study sample. Additionally, Bunod et al. [28] reported no manifestations of glaucoma among the 24 patients studied.

#### 4.4.7. Amyloid Deposits in the Lens

The presence of amyloid deposits in the lens was reported by six out of sixteen authors (37.5%). The average frequency of this manifestation was 25.4%, with a standard deviation of 10.8%.

#### 4.4.8. Retinal Hemorrhage

The frequency of retinal hemorrhage was reported by only 3 out of sixteen authors (18.8%). The average frequency of this manifestation was 17.0%, with a standard deviation of 9.36%.

#### 4.4.9. Vascular Tortuosity

Vascular tortuosity was reported by three out of sixteen studies (18.8%). It is noteworthy that in Ando et al., the term “tortuosity” was not used; instead, “abnormal conjunctival vessels” were described, observed in 100% of the patients during follow‐up. The average frequency of vascular tortuosity was 39%, with a standard deviation of 43.3%.

#### 4.4.10. Conjunctival Lymphangiectasia

Finally, conjunctival lymphangiectasia was reported by only one study [28], with a frequency of 54.2%.

### 4.5. Qualitative Description of Relevant Ophthalmological Findings

The case series by Beirão et al. [17] evaluated over 500 patients with ATTRv (Val30Met) and demonstrated that among the most common ocular manifestations, an abnormal tear BUT was the most prevalent (79.5%). When differentiating between anterior and posterior segment manifestations, the primary anterior manifestation was amyloid deposition in the iris (38.4%), while the main posterior manifestation was glaucoma (20%). In this study, patient sex did not correlate with the presented manifestations. Abnormalities in conjunctival vessels were observed with a higher incidence in the intermediate‐onset group (17.2%), followed by the early‐onset group (15.6%) and least frequently in the late‐onset group (4%). Early onset was defined as < 40 years, late onset as > 50 years, with intermediate onset occurring between these two extremes. The evaluation of the Schirmer test indicated a higher frequency of abnormalities in the late‐onset group (80.6%), followed by the intermediate‐onset group (71.9%) and the early‐onset group (63.6%). Additionally, the frequency of all manifestations increased with disease progression. Another association found in this study was the positive correlation between glaucoma and serrated iris (92.1% of eyes with glaucoma exhibited serrated iris, *p* < 0.001), as well as between retinal amyloid angiopathy and vitreous amyloidosis (69% of eyes with angiopathy showed vitreous amyloidosis, *p* < 0.001) [17].

It is also worth mentioning that the study by Marques et al. which evaluated 24 eyes of patients with ATTRv (Val30Met), concluded that the presence of serrated iris appears to be associated with more advanced subclinical microangiopathic retinopathy compared to eyes without iris changes [29].

In the study by Liu et al. [23], conducted on the eyes of a Chinese family with the ATTRvG83A variant, other ocular manifestations identified included secondary glaucoma, dry eye, and pupillary anomalies, all of which were less prevalent than vitreous opacities [23].

Regarding retinal changes, the study by Rousseau et al. [27] aimed to describe the patterns of involvement in amyloid retinopathy and choroidal angiopathy. Fluorescein angiography was performed on 16 patients with the Val30Met variant, all exhibiting symptoms of polyneuropathy, and microaneurysms were found to be the main alteration in retinopathy, present in all affected eyes. Additionally, retinal hemorrhages, focal retinal obstruction without visible emboli, and neovascular glaucoma were also identified. For choroidopathy, late hypercyanescence along the choroidal arteries in the central and mid‐peripheral fundus was observed in all patients, regardless of the presence or absence of other intraocular manifestations of amyloidosis. It is noteworthy that hypercyanescence exhibited three main patterns: “fireworks,” disseminated, and punctate. Furthermore, this study also observed a coexistence of retinopathy with vitreous opacities [27].

Vitreous opacities were also identified as the primary ocular manifestation in patients with the Glu89Lys, Gly47Arg, and Gly6Ser variants in the studies by Reynolds et al. and Ruiz‐Medrano et al. [26, 31]. Contrary to the trend of having this alteration as the main one, the study by Ando et al. [18] found that only 16.2% of patients exhibited vitreous opacities by the end of follow‐up, whereas pupillary abnormalities were observed in nearly 73% of patients during the same period. In this study, pupillary changes and conjunctival vessel abnormalities (86.4%) were the most prevalent manifestations.

Glaucoma, on the other hand, was the least prevalent manifestation, identified in 5.4% of the 37 patients included in the study. It is noteworthy that an increase in the frequency of ophthalmological manifestations was observed over time, with 100% of patients exhibiting pupillary changes by the end of the study period. Furthermore, the progression of ophthalmological manifestations followed a specific order: initially conjunctival vessel abnormalities, followed by dry eye, pupillary disturbances, vitreous opacities, and glaucoma [18].

Regarding glaucoma, the study by Kimura et al. [19] reported that this manifestation was significantly less prevalent in patients with the Val30Met variant compared to other variants found in the studied patients. This study also identified a statistically significant relationship between amyloid deposition in the pupil and glaucoma, as well as vitreous opacities. It is important to note that other ophthalmological manifestations with significant frequency were observed, including abnormal conjunctival vessels (61% of patients), keratoconjunctivitis sicca (60%), vitreous opacities (35%), amyloid deposition in the pupil (31%), and wavy pupil (8%) [19].

The study by Choi et al. [30], a review, uniquely demonstrated ocular involvement in patients with the Asp38Ala and Thr59Lys mutations, showing a higher frequency of retinal deposits and lens opacities [30].

Although there is no consensus among the studies, a higher frequency and ophthalmological involvement have been observed in female patients, particularly in the studies by Reynolds et al. and Choi et al. [26, 30].

Regarding ethnicity, the study by Kawaji et al. [20] compared the ophthalmological manifestations exhibited by Caucasian versus Asian patients with the Val30Met variant, finding no statistically significant differences in the onset time of polyneuropathy and vitreous opacities. The only differences observed between the populations were a later and more frequent involvement among Caucasians compared to Asians [20].

In the study by Liu et al. [23], a patient with ATTRv (Gly83Arg mutation) who was not transplanted required laser cyclophotocoagulation after failing to achieve satisfactory intraocular pressure levels with medical therapy and trabeculectomy [23]. Similarly, Kimura reported difficulties in controlling intraocular pressure despite medical and surgical interventions, leading to complications such as neovascular glaucoma [19]. These findings suggest that glaucoma secondary to amyloidosis may represent a condition that is challenging to manage.

In contrast to the previous inference regarding glaucoma, the surgical management of vitreous opacities through vitrectomy appears to be successful. The study by Zou et al. reports cases of patients with ATTR (Ala36Pro variant) who experienced rapidly progressive vision loss and showed significant improvement in visual acuity following surgery [24]. The study by Long et al. supports these findings [22]. It is important to emphasize, however, that these studies were considered to be at high risk of bias, so inferences drawn from their data should be analyzed with caution.

Finally, in the search for disease markers, the study by Bunod et al. [28] evaluated the ability of conjunctival lymphangiectasia to predict systemic disease severity in patients with the Ser77Tyr variant. It demonstrated a positive predictive value (PPV) of 84.6% and a negative predictive value (NPV) of 81.8% for severe functional impairment. Additionally, the PPV was 92.3% and the NPV was 72.7% for amyloid cardiomyopathy [28].

### 4.6. Qualitative Description of Relevant Information on Medical and Surgical Management

The study by Beirão et al. [25] analyzed ocular manifestations in patients diagnosed with ATTRv (Val30Met mutation) over periods of 5, 10, and 15 years. It demonstrated an increase in the frequency of all manifestations over time, despite liver transplantation, when compared to the group without transplantation. Furthermore, compared to the control group, liver transplantation was associated with worsening effects regarding amyloid fibril deposition in the anterior capsule, vitreous, and glaucoma. In contrast, for iris deposition, tear BUT alterations, and retinal angiopathy, transplantation did not appear to influence the progression times of the disease assessed [25]. This finding was also observed in another study, such as that by Buxbaum et al., which was based on a subgroup analysis. In this study, the frequency of ocular manifestations was higher in patients who underwent liver transplantation compared to those who only received supportive therapy, after 10 years of diagnosis [16].

Furthermore, supporting these findings, the case series by de Beirão et al. [17] demonstrated a higher frequency of amyloid deposition in the iris, dentate iris, anterior capsule of the lens, and vitreous amyloidosis in transplanted patients compared to those who were not transplanted, with statistical significance noted (42.9% vs. 26.9%, *p* < 0.001; 21.6% vs. 30.3%, *p* < 0.05; 23.9% vs. 36.1%, *p* = 0.009; 11.2% vs. 19.8%, *p* < 0.025, respectively) [17].

Additionally, current data suggest that liver transplantation does not appear to be beneficial for treating the ocular manifestations of TTR amyloidosis. Studies have shown that not only does transplantation fail to address ophthalmic diseases, but it may also be associated with a potential long‐term worsening of these conditions [16, 21, 25].

In line with this information, Buxbaum et al. reported that the use of tafamidis 20 mg/day after 5 years of diagnosis did not result in a statistically significant difference compared to supportive therapy regarding the frequency of ocular manifestations over time, although it did show a difference compared to liver transplantation [16].

### 4.7. Correlation Between Ocular and Systemic Manifestations

It was not possible to establish correlations between ocular manifestations and systemic conditions such as polyneuropathy and heart failure due to methodological heterogeneity and the lack of relevant data in the studies reviewed. The study that performed an analysis correlating ocular manifestations with systemic conditions was that of Bunod et al., which sought disease markers. This study concluded that, for patients with the Ser77Tyr mutation, the presence of conjunctival lymphangiectasia has a PPV of 92.3% and a NPV of 72.7% for amyloid cardiomyopathy, as well as PPV and NPV of 84.6% and 81.8%, respectively, for severe functional impairment. Although it was one of the studies considered to have a low risk of bias, being the only one to conduct such analyses, more accurate inferences cannot be drawn solely from its findings.

## 5. Discussion

After analyzing the included studies, comprising a sample of 1792 patients, significant variation was observed in the frequency of different ocular manifestations. The frequency of vitreous opacities was assessed in 87.5% of the studies, with an overall average of 37.3%. However, the analysis revealed high variability among the studies, evidenced by a significant standard deviation of 32.1%, highlighting the need to interpret these results with caution. The presence of dry eye was documented in 50% of the reviewed studies, with an average frequency of 47.8%. This average was influenced by specific studies that reported divergent results, resulting in a standard deviation of 33.4%. The pupillary manifestation known as “shell pupil” or “wavering pupil” was reported in 37.5% of the studies, with an average frequency of 35.5% and a standard deviation of 33.6%.

The frequency of glaucoma varied considerably, with an overall average of 18% and a standard deviation of 15.7%. The presence of amyloid deposits in the lens was observed in 37.5% of the studies, with an average frequency of 25.4% and a standard deviation of 10.8%. Retinal hemorrhage was a less frequent manifestation, documented in only 18.8% of the studies, with an average frequency of 17.0% and a standard deviation of 9.36%.

Vascular tortuosity was also mentioned in 18.8% of the studies, although there was variation in the terminology used among the articles, with a frequency of 39% and a standard deviation of 43.3%. Finally, conjunctival lymphangiectasia was a rare finding, reported in a single study with a frequency of 54.2%. Additionally, the most prevalent TTR gene mutation was Val30Met, accounting for approximately 92% of the observations (1643 cases) in the analyzed studies.

This systematic review is notable for its meticulous approach to analyzing the ophthalmological manifestations of hereditary TTR amyloidosis. However, it is essential to acknowledge the inherent limitations of the included studies, which predominantly consist of case series, resulting in limited statistical power.

Despite over 43% of the included studies being classified as low risk of bias, a positive indicator of evidence quality, the presence of medium‐ and high‐risk studies must be taken into account, as they can introduce uncertainties or limitations to their conclusions.

Additionally, it is important to note that the studies included in this work varied significantly in design, population, and outcome assessment methods and not always provide numerical data. As a result, it was not possible to conduct a meta‐analysis as recommended by the PRISMA checklist, which also complicated the use of the NOS. Since some questions of the scale could not be answered by the studies themselves, “false negatives” would be generated in the bias risk analysis.

In an effort to reduce bias in this analysis, a modified scale was chosen, deemed satisfactory by the researchers for the situation, as it considers the same parameters as the NOS in a more concise manner.

It is worth noting that the bias risk analysis revealed a significant number of studies with follow‐ups deemed unsatisfactory or insufficient, indicating that this aspect needs improvement in future research.

Another point to consider is that most articles had small patient samples, limiting the ability to perform meaningful statistical associations or inferences. A high standard deviation, especially one close to the mean, suggests significant variability in the ocular manifestation in question. This variability may be attributed to various factors, including methodological differences. Nonetheless, the descriptive analysis of the results is valid and can assist future studies.

Additionally, the scarcity of Brazilian publications on the subject raises concerns about the representativeness and generalizability of the results for the Brazilian population, which may exhibit distinct phenotypic characteristics due to our ethnic diversity. This gap in knowledge underscores the importance of encouraging and fostering ophthalmological research within the national context, aiming for a more comprehensive understanding of ATTR manifestations and, consequently, improvements in diagnostic and treatment protocols for affected patients.

Due to the significant variability in study design, population, outcome assessment methods, and the inconsistent provision of numerical data among the included studies, it was not possible to conduct a meta‐analysis as recommended by the PRISMA checklist.

Therefore, although the findings of this systematic review provide valuable insights into the ocular manifestations of ATTRv, and there are no other review offering detailed frequency analyses like this one, it is imperative to interpret them with caution due to the methodological limitations of the analyzed studies and the lack of representativeness of the Brazilian population.

## 6. Conclusion

Vitreous opacities were the most prevalent ophthalmological manifestations, followed by dry eye and wavering pupils. However, due to the identified limitations, there remains a continuous need for research in this field to address knowledge gaps and improve the quality of ophthalmic care provided to patients with ATTRv.

## Author Contributions

All named authors are confirmed, and all agree to be accountable for the research presented:

⁃ Claudia Pedreira: conceptualization, investigation, supervision, resources, and writing–review and editing.
⁃ Maria Luisa Fialho: writing–original draft, review, and editing.
⁃ Juliana Marback: writing–original draft, review, and editing.
⁃ Luiz Eduardo Ritt: conceptualization, project administration, supervision, and writing–review and editing.
⁃ Marcela Costa: writing–review and editing and validation.
⁃ Tonnison Silva: writing–review and editing and validation.

The order of authors listed in the manuscript has also been approved.

## Funding

This study had no funding.

## Disclosure

The authors are solely responsible for the content and writing of the article. All translated content, interpretations, and the final version of the manuscript were thoroughly reviewed, verified, and approved by the authors.

## Conflicts of Interest

The authors declare no conflicts of interest.

## Supporting Information

Additional supporting information can be found online in the Supporting Information section.

Table 1 of the Supporting Information presents the detailed electronic search strategy used for all databases included in this systematic review. Table 2 provides the completed PRISMA 2020 checklist, outlining adherence to the reporting standards recommended for systematic reviews.

## Supporting information


**Supporting Information 1** Table 1 of Supporting Information—Detailed Search Strategy.

**Supporting Information 2** Table 2 of Supporting Information—PRISMA Checklist.

## Data Availability

Data sharing is not applicable to this article as no datasets were generated or analyzed during the current study.
