# Supplementary material for: Ophthalmological Manifestations of Hereditary Amyloidosis due to Transthyretin: A Systematic Review
Source: J Ophthalmol. 2026 Apr 30;2026:5277348. doi: 10.1155/joph/5277348 (PMC13131319; doi:10.1155/joph/5277348)
Supplement: Supplementary file 1 — Supporting Information 1 Table 1 of Supporting Information—Detailed Search Strategy. [file JOPH-2026-5277348-s002.docx]

**Supplementary Files**

Table 1 of the Supplementary Files presents the detailed electronic search strategy used for all databases included in this systematic review. Table 2 provides the completed PRISMA 2020 checklist, outlining adherence to the reporting standards recommended for systematic reviews.

**[1]** Table 1 of Supplementary Files - Detailed Search Strategy

| platform | search strategy | number of results |
| --- | --- | --- |
| pubmed | ("amyloidosis familial"[Title] OR (("Amyloidosis"[MeSH Terms] OR  "Amyloidosis"[All Fields] OR "amyloidoses"[All Fields]) AND  "Hereditary"[Title/Abstract]) OR "amyloidosis hereditary"[Title/Abstract] OR  "amyloidosis, familial"[MeSH Terms] OR "amyloidosis, familial"[MeSH Terms] OR  "transthyretin amyloidosis"[Title/Abstract] OR (("Hereditary"[All Fields] AND  ("prealbumin"[MeSH Terms] OR "prealbumin"[All Fields] OR "Transthyretin"[All  Fields] OR "transthyretins"[All Fields])) AND "Amyloidosis"[MeSH Terms])) AND  ("Glaucoma"[MeSH Terms] OR "Glaucoma"[Title/Abstract] OR  "Cataract"[Title/Abstract] OR "Cataract"[MeSH Terms] OR "lens  opacity"[Title/Abstract] OR “Vitreous opacity” [Title/Abstract] OR “Pupil abnormality” [Title/Abstract] OR “Dry eyes” [Title/Abstract] OR “keratoconjunctivitis sicca” [Title/Abstract]). | 53 |
| embase | ('familial amyloidosis'/exp OR 'familial amyloidosis') AND 'ocular manifestations' | 25 |
| scopus | ( TITLE-ABS-KEY ( *amyloidosis, AND familial* ) OR TITLE-ABS-  KEY ( *amyloidoses, AND hereditary* ) OR TITLE-ABS-  KEY ( *transthyretin AND amyloidosis* ) OR TITLE-ABS-  KEY ( hereditary AND transthyretin AND amyloidosis ) AND TITLE-ABS-  KEY ( *ocular AND manifestations* ) ) | 59 |
| web of science | Amyloidosis, Familial (All Fields) OR Amyloidoses, Hereditary (All Fields) OR Hereditary  transthyretin amyloidosis (All Fields) AND Ocular Manifestations (All Fields) | 14 |

**[2]** Table 2 of Supplementary Files – PRISMA Checklist

| **Section and Topic** | **Item #** | **Checklist item** | **Location where item is reported** |
| --- | --- | --- | --- |
| **TITLE** | | |  |
| Title | 1 | Identify the report as a systematic review. | Pages 01, 04 and 07 |
| **ABSTRACT** | | |  |
| Abstract | 2 | See the PRISMA 2020 for Abstracts checklist. | Page 04 |
| **INTRODUCTION** | | |  |
| Rationale | 3 | Describe the rationale for the review in the context of existing knowledge. | Pages 05-07 |
| Objectives | 4 | Provide an explicit statement of the objective(s) or question(s) the review addresses. | Pages 07-08 |
| **METHODS** | | |  |
| Eligibility criteria | 5 | Specify the inclusion and exclusion criteria for the review and how studies were grouped for the syntheses. | Pages 07-09; “Study Question” and “Study selection” in “Methods” |
| Information sources | 6 | Specify all databases, registers, websites, organisations, reference lists and other sources searched or consulted to identify studies. Specify the date when each source was last searched or consulted. | Pages 08-09; “Study selection” in “Methods”. The date when each source was las consulted was not registered. |
| Search strategy | 7 | Present the full search strategies for all databases, registers and websites, including any filters and limits used. | Page 08; “Search strategy” in “Methods”. |
| Selection process | 8 | Specify the methods used to decide whether a study met the inclusion criteria of the review, including how many reviewers screened each record and each report retrieved, whether they worked independently, and if applicable, details of automation tools used in the process. | Pages 08-09; “Study selection” in “Methods”. The date when each source was las consulted was not registered. |
| Data collection process | 9 | Specify the methods used to collect data from reports, including how many reviewers collected data from each report, whether they worked independently, any processes for obtaining or confirming data from study investigators, and if applicable, details of automation tools used in the process. | Page 09; “Data extraction” in “Methods”. |
| Data items | 10a | List and define all outcomes for which data were sought. Specify whether all results that were compatible with each outcome domain in each study were sought (e.g. for all measures, time points, analyses), and if not, the methods used to decide which results to collect. | Pages 08-09; “Study selection” and “Data extraction” in “Methods”. |
|  | 10b | List and define all other variables for which data were sought (e.g. participant and intervention characteristics, funding sources). Describe any assumptions made about any missing or unclear information. | Pages 08-09; “Study selection” and “Data extraction” in “Methods”. |
| Study risk of bias assessment | 11 | Specify the methods used to assess risk of bias in the included studies, including details of the tool(s) used, how many reviewers assessed each study and whether they worked independently, and if applicable, details of automation tools used in the process. | Pages 09-10; “Bias risk analysis” in “Methods”. |
| Effect measures | 12 | Specify for each outcome the effect measure(s) (e.g. risk ratio, mean difference) used in the synthesis or presentation of results. | Page 09; “Data extraction” in “Methods”. |
| Synthesis methods | 13a | Describe the processes used to decide which studies were eligible for each synthesis (e.g. tabulating the study intervention characteristics and comparing against the planned groups for each synthesis (item #5)). | Pages 07-10; “Study Question”, “Study selection” and “Bias risk analysis” in “Methods” |
|  | 13b | Describe any methods required to prepare the data for presentation or synthesis, such as handling of missing summary statistics, or data conversions. | Page 09; “Data extraction” in “Methods”. |
|  | 13c | Describe any methods used to tabulate or visually display results of individual studies and syntheses. | Page 09; “Data extraction” in “Methods”. |
|  | 13d | Describe any methods used to synthesize results and provide a rationale for the choice(s). If meta-analysis was performed, describe the model(s), method(s) to identify the presence and extent of statistical heterogeneity, and software package(s) used. | Page 09; “Data extraction” in “Methods”. |
|  | 13e | Describe any methods used to explore possible causes of heterogeneity among study results (e.g. subgroup analysis, meta-regression). | Not applicable |
|  | 13f | Describe any sensitivity analyses conducted to assess robustness of the synthesized results. | Not applicable |
| Reporting bias assessment | 14 | Describe any methods used to assess risk of bias due to missing results in a synthesis (arising from reporting biases). | Pages 09-10; “Bias risk analysis” in “Methods”. |
| Certainty assessment | 15 | Describe any methods used to assess certainty (or confidence) in the body of evidence for an outcome. | Not applicable |
| **RESULTS** | | |  |
| Study selection | 16a | Describe the results of the search and selection process, from the number of records identified in the search to the number of studies included in the review, ideally using a flow diagram. | Pages 11-12; “Study selection” in Results. |
|  | 16b | Cite studies that might appear to meet the inclusion criteria, but which were excluded, and explain why they were excluded. | Pages 11-12; “Study selection” in Results. |
| Study characteristics | 17 | Cite each included study and present its characteristics. | Pages 13-23 |
| Risk of bias in studies | 18 | Present assessments of risk of bias for each included study. | Pages 13-14; “Bias risk analysis” in Results”. |
| Results of individual studies | 19 | For all outcomes, present, for each study: (a) summary statistics for each group (where appropriate) and (b) an effect estimate and its precision (e.g. confidence/credible interval), ideally using structured tables or plots. | Pages 13-14; “Bias risk analysis” in Results”. |
| Results of syntheses | 20a | For each synthesis, briefly summarise the characteristics and risk of bias among contributing studies. | Pages 13-14; “Bias risk analysis” in Results”. |
|  | 20b | Present results of all statistical syntheses conducted. If meta-analysis was done, present for each the summary estimate and its precision (e.g. confidence/credible interval) and measures of statistical heterogeneity. If comparing groups, describe the direction of the effect. | Pages 14-18; “Summary of individual study results” in “Results”. |
|  | 20c | Present results of all investigations of possible causes of heterogeneity among study results. | Not applicable |
|  | 20d | Present results of all sensitivity analyses conducted to assess the robustness of the synthesized results. | Not applicable |
| Reporting biases | 21 | Present assessments of risk of bias due to missing results (arising from reporting biases) for each synthesis assessed. | Not applicable |
| Certainty of evidence | 22 | Present assessments of certainty (or confidence) in the body of evidence for each outcome assessed. | Not applicable |
| **DISCUSSION** | | |  |
| Discussion | 23a | Provide a general interpretation of the results in the context of other evidence. | Pages 23-26 |
|  | 23b | Discuss any limitations of the evidence included in the review. | Page 25 |
|  | 23c | Discuss any limitations of the review processes used. | Pages 25-26 |
|  | 23d | Discuss implications of the results for practice, policy, and future research. | Page 26 |
| **OTHER INFORMATION** | | |  |
| Registration and protocol | 24a | Provide registration information for the review, including register name and registration number, or state that the review was not registered. | Not registered |
|  | 24b | Indicate where the review protocol can be accessed, or state that a protocol was not prepared. | Protocol was not prepared |
|  | 24c | Describe and explain any amendments to information provided at registration or in the protocol. | Not applicable |
| Support | 25 | Describe sources of financial or non-financial support for the review, and the role of the funders or sponsors in the review. | Page 27; “Funding statement”. |
| Competing interests | 26 | Declare any competing interests of review authors. | Page 26; “Conflict of interest”. |
| Availability of data, code and other materials | 27 | Report which of the following are publicly available and where they can be found: template data collection forms; data extracted from included studies; data used for all analyses; analytic code; any other materials used in the review. | Not publicly available |

*From:*  Page MJ, McKenzie JE, Bossuyt PM, Boutron I, Hoffmann TC, Mulrow CD, et al. The PRISMA 2020 statement: an updated guideline for reporting systematic reviews. BMJ 2021;372:n71. doi: 10.1136/bmj.n71
